# Use of Loop Diuretics is Associated with Increased Mortality in Patients with Suspected Coronary Artery Disease, but without Systolic Heart Failure or Renal Impairment: An Observational Study Using Propensity Score Matching

**DOI:** 10.1371/journal.pone.0124611

**Published:** 2015-06-01

**Authors:** Hall Schartum-Hansen, Kjetil H. Løland, Gard F. T. Svingen, Reinhard Seifert, Eva R. Pedersen, Jan E. Nordrehaug, Øyvind Bleie, Marta Ebbing, Christ Berge, Dennis W. T. Nilsen, Ottar Nygård

**Affiliations:** 1 Department of Heart Disease, Haukeland University Hospital, Bergen, Norway; 2 Section for Cardiology, Department of Clinical Science, University of Bergen, Bergen, Norway; 3 Norwegian Institute of Public Health, Bergen, Norway; 4 Stavanger University Hospital, Stavanger, Norway; University of Naples Federico II, ITALY

## Abstract

**Background:**

Loop diuretics are widely used in patients with heart and renal failure, as well as to treat hypertension and peripheral edema. However, there are no randomized, controlled trials (RCT) evaluating their long term safety, and several observational reports have indicated adverse effects. We sought to evaluate the impact of loop diuretics on long term survival in patients with suspected coronary artery disease, but without clinical heart failure, reduced left ventricular ejection fraction or impaired renal function.

**Method and Findings:**

From 3101 patients undergoing coronary angiography for suspected stable angina pectoris, subjects taking loop diuretics (n=109) were matched with controls (n=198) in an attempted 1:2 ratio, using propensity scores based on 59 baseline variables. During median follow-up of 10.1 years, 37.6% in the loop diuretics group and 23.7% in the control group died (log-rank p-value 0.005). Treatment with loop diuretics was associated with a hazard ratio (95% confidence interval) of 1.82 (1.20, 2.76), and the number needed to harm was 7.2 (4.1, 30.3). Inclusion of all 3101 patients using propensity score weighting and adjustment for numerous covariates provided similar estimates. The main limitation is the potential of confounding from unmeasured patient characteristics.

**Conclusions:**

The use of loop diuretics in patients with suspected coronary artery disease, but without systolic heart failure or renal impairment, is associated with increased risk of all-cause mortality. Considering the lack of randomized controlled trials to evaluate long term safety of loop diuretics, our data suggest caution when prescribing these drugs to patients without a clear indication.

## Introduction

Loop diuretics are widely used drugs, constituted mainly by the sulfonamide derivates furosemide, bumetanide and torsemide, which act on the thick ascending limb of the loop of Henle. They are primarily prescribed to patients with acute and chronic heart failure with signs of fluid overload, but also used in the treatment of acute and chronic renal failure, hepatic failure, hypertension and peripheral edema. Loop diuretics have been a pillar in the treatment of systolic heart failure for decades, and are strongly recommended in the current heart failure guidelines from both the European Society of Cardiology and the American College of Cardiology Foundation/American Heart Association [1, 2]. However, no large randomized, controlled trials (RCTs) have been conducted to ensure long term efficacy and safety [3–5], whereas several observational studies have associated long term treatment with poor outcome in patients with systolic heart failure [6–12]. Epidemic overuse and substantial toxicity have been described, particularly among the elderly [13, 14]. The large scale use is confirmed by the high prescription rate in Norway (24.9/1000 of the total population, and 178/1000 among those aged 80–84 in 2012) [15]. Thus, there is reason to assume that a considerable number of patients are treated with loop diuretics where no or other medications would be superior.

The aim of the current study was to assess the relation between loop diuretics and mortality in a cohort of suspected stable coronary artery disease (CAD) patients without systolic heart failure or renal failure, i.e. patients most likely without a strong indication for receiving the drug, using propensity score matching to reduce confounding.

## Methods

### Study population

The study population consisted of 4164 patients with suspected stable angina who underwent coronary angiography at Haukeland University Hospital and Stavanger University Hospital, Norway, from 1999 to 2004. A total of 2573 (61.8%) of these patients were included in the Western Norway B Vitamin Intervention Trial (WENBIT) [16]. We excluded 718 patients with either left ventricular ejection fraction (LVEF) <50%, a history of unspecified or right heart failure, estimated glomerular filtration rate (eGFR) <60 mL/min/1.73 m^2^, aortic stenosis or liver failure. Of the remaining 3446, 3101 had complete datasets with regard to all the covariates we planned to use for propensity score matching. Of these, 126 patients were using loop diuretics at discharge following the initial evaluation. Written, informed consent was obtained from all participants. The study was approved by the Regional Committee for Medical and Health Research Ethics, the Data Inspectorate, and the Norwegian Directorate of Health.

### Data collection, biochemical analyses and follow-up

Information on patients’ lifestyle and medical history was obtained from self-administered questionnaires and verified by comparing to hospital records. The records of patients registered as taking loop diuretics at baseline were additionally validated. Diabetes included both type 1 and 2. LVEF was obtained either by echocardiography or by ventriculography performed during cardiac catheterization. Smokers included self-reported current smokers, those reporting having quit within the last 4 weeks and subjects with plasma cotinine >85 nmol/L. A more detailed characterization has been published previously [17]. Plasma was usually sampled a few days before coronary angiography, and samples were prepared and immediately frozen at -80°C. The patients were followed up through December 31, 2012 and the outcome data were obtained by linking the unique personal identification numbers to the Cause of Death Registry of Norway. Data on changes in drug prescriptions and patient compliance during follow-up were not available.

### Statistical analyses

Based on 59 baseline variables, including anthropometric, clinical and biochemical data, the propensity score for loop diuretic usage was calculated using a logistic regression model for each of the 3101 patients [18]. Patients receiving loop diuretics (treated) and controls were matched on the logit of the propensity score, using calipers of width equal to 0.2 times the standard deviation of the logit of the propensity score. We used nearest neighbour matching attempting a 1:2 ratio, with no interactions included. 17 patients on loop diuretics could not be matched (S1 Fig). Balance between the treatment and control groups was assessed by unweighted standardized mean differences, variance ratios between treated and controls, histograms and jitter plots of propensity score distribution and visual inspection of QQ plots. The null hypothesis of equality of variances for continuous variables was estimated following an F-distribution with 107 and 195 degrees of freedom [19]. The 2.5 and 97.5 percentiles were 0.61 and 1.39, respectively. Post-matching continuous variables are shown as means (standard deviation [SD]) and medians (interquartile range [IQR]) and categorical variables as percentages. Statistical differences between the groups were tested with independent T-tests, Wilcoxon signed-rank tests and Chi-square tests. All-cause mortality was initially explored with a Kaplan-Meier plot and log-rank test. Number needed to harm was calculated as the inverse of the attributable risk (incidence of death in treated minus controls) and the 95% confidence intervals (CI) by using the simple Wald method. Cox regression analyses were conducted to calculate hazard ratios (HRs), both using crude data and extensive adjustment for covariates. Not all matching variables were included in these analyses, in order to avoid collinearity (e.g. hypertension but not systolic blood pressure). A time dependent covariate was used to test for non-proportional hazards. Propensity score weighted Cox regression analyses were performed in the entire cohort of 3101 patients in a similar manner. In order to assess the magnitude of potential residual confounding, sensitivity analyses were performed using the method described by Lin et al [20]. All analyses and plots were done using R (version 3.1.1). For propensity matching and weighting we used the MatchIt [21, 22] and Twang packages, respectively.

## Results

### Baseline characteristics

109 matched pairs were formed, of which 89 pairs consisted of one patient receiving loop diuretics and two controls, and 20 pairs consisting of one patient using loop diuretics and one control. Table 1 shows the post-matching baseline characteristics of the treatment and control groups. The mean (SD) age was 64.9 (9.8) years and 55.0% were males. The most prevalent pre-existing medical conditions were hypertension (64.8%) and acute myocardial infarction (AMI) (47.2%), and almost three quarters had angiographic evidence of CAD. About three quarters were treated with beta-blockers, statins and aspirin. Baseline characteristics before matching are shown in S1 Table.

**Table 1:** Baseline characteristics after matching.

		Controls	Loop diuretics	P-value
N		198	109	
Age (years)		64.9 (9.2)	65 (11)	0.95
Sex (male %)		114 (57.6)	55 (50.5)	0.28
WENBIT participation (%)		95 (48)	57 (52.3)	0.55
Smoking (%)		47 (23.7)	31 (28.4)	0.44
*Medical history (%)*				
Hypertension		124 (62.6)	75 (68.8)	0.34
Diabetes		25 (12.6)	16 (14.7)	0.74
Family history of CAD		58 (29.3)	35 (32.1)	0.70
Acute myocardial infarction		93 (47)	52 (47.7)	1.00
PCI		34 (17.2)	23 (21.1)	0.49
CABG		23 (11.6)	13 (11.9)	1.00
Peripheral vascular disease		20 (10.1)	13 (11.9)	0.76
Cerebrovascular disease		30 (15.2)	17 (15.6)	1.00
Active cancer		4 (2)	3 (2.8)	0.99
Cured cancer		12 (6.1)	10 (9.2)	0.44
DVT or vein surgery		8 (4)	5 (4.6)	1.00
Pulmonary disease		45 (22.7)	27 (24.8)	0.79
Kidney disease		2 (1)	1 (0.9)	1.00
Atrial fibrillation		32 (16.2)	19 (17.4)	0.90
*Clinical and paraclinical findings*				
Dyspnea (NYHA class) (%)				
	0–1	116 (58.6)	62 (56.9)	0.87
	2	63 (31.8)	34 (31.2)	1.00
	3	19 (9.6)	13 (11.9)	0.66
	4	0	0	
Body mass index (kg/m2)		28.2 (5.3)	28.4 (5)	0.76
ECG rythm (sinus %)		178 (89.9)	96 (88.1)	0.76
LVEF (%)		66.4 (8.1)	65.8 (8.8)	0.54
Angiographic extent of CAD (%)				
	0-vessel disease	60 (30.3)	31 (28.4)	0.83
	1-vessel disease	46 (23.2)	23 (21.1)	0.78
	2-vessel disease	46 (23.2)	30 (27.5)	0.49
	3-vessel disease	46 (23.2)	25 (22.9)	1.00
Systolic BP (mmHg)		143 (19)	143 (21)	0.95
Diastolic BP (mmHg)		80.9 (10)	81.2 (11)	0.82
*Blood parameters*				
eGFR (mL/min/1.73 m2)		85.5 (13)	84.9 (15)	0.76
Uric acid (umol/L)		386 (85)	388 (96)	0.88
Hemoglobin (g/dL)		14.1 (1.3)	13.9 (1.3)	0.21
Potassium (mmol/L)		4.24 (0.32)	4.23 (0.32)	0.72
Sodium (mmol/L)		142 (2.4)	142 (2.3)	0.52
C-reactive protein (mg/L)		2.2 (3.8)	3.06 (3.6)	0.04
HbA1c (%)		6.1 (1.3)	6.17 (1.2)	0.63
Glucose (mmol/L)		248 (60)	257 (66)	0.28
Platelet count (10^9/L)		6.41 (2.4)	6.6 (2.1)	0.47
WBC (10^9/L)		7.41 (2.2)	7.5 (1.9)	0.72
Triglycerides (mmol/L)		1.52 (1.1)	1.4 (0.86)	0.98
Low density lipoprotein (mmol/L)		3.04 (0.95)	3.07 (1.1)	0.80
Apolipoprotein A1 (g/L)		1.37 (0.28)	1.38 (0.29)	0.69
Apolipoprotein B (g/L)		0.892 (0.23)	0.897 (0.24)	0.86
Troponin T (ng/L)		6 (9.8)	7 (10)	0.75
*Discharge medication (%)*				
Aspirin		152 (76.8)	84 (77.1)	1.00
ADP-receptor inhibitor		24 (12.1)	11 (10.1)	0.73
Warfarin		17 (8.6)	12 (11)	0.62
ACEI or/and ARB		101 (51)	57 (52.3)	0.92
Beta-blocker		150 (75.8)	82 (75.2)	1.00
Digoxin		15 (7.6)	11 (10.1)	0.59
Spironolactone		5 (2.5)	2 (1.8)	1.00
Thiazide		18 (9.1)	8 (7.3)	0.75
Calcium antagonist		57 (28.8)	38 (34.9)	0.33
Nitrate		55 (27.8)	34 (31.2)	0.62
Statin		148 (74.7)	86 (78.9)	0.50
Insulin		7 (3.5)	4 (3.7)	1.00
Metformin		12 (6.1)	8 (7.3)	0.85
Sulfonylurea		11 (5.6)	6 (5.5)	1.00
COPD-medication		28 (14.1)	16 (14.7)	1.00
NSAID		10 (5.1)	6 (5.5)	1.00
Corticosteroid		13 (6.6)	7 (6.4)	1.00
Antidepressant		16 (8.1)	9 (8.3)	1.00
Antipsychotic		2 (1)	2 (1.8)	0.93

Continuous variables are shown as means (standard deviation) and medians (interquartile range) and categorical variables as numbers (percentage). Abbreviations: WENBIT = WEstern Norway B-vitamin Trial; CAD = coronary artery disease; PCI = percutaneous coronary intervention; CABG = coronary artery bypass graft; PVD = peripheral vascular disease; DVT = deep venous thrombosis; NYHA = New York Heart Association; BP = blood pressure; eGFR = estimated glomerular filtration rate; HbA1c = glycated hemoglobin; WBC = white blood cell count; ACEI = angiotensin converting enzyme inhibitor; ARB = angiotensin receptor blocker; COPD = chronic obstructive pulmonary disease; NSAID = non-steroid anti-inflammatory drug.

### Balance analyses

**Fig 1:**
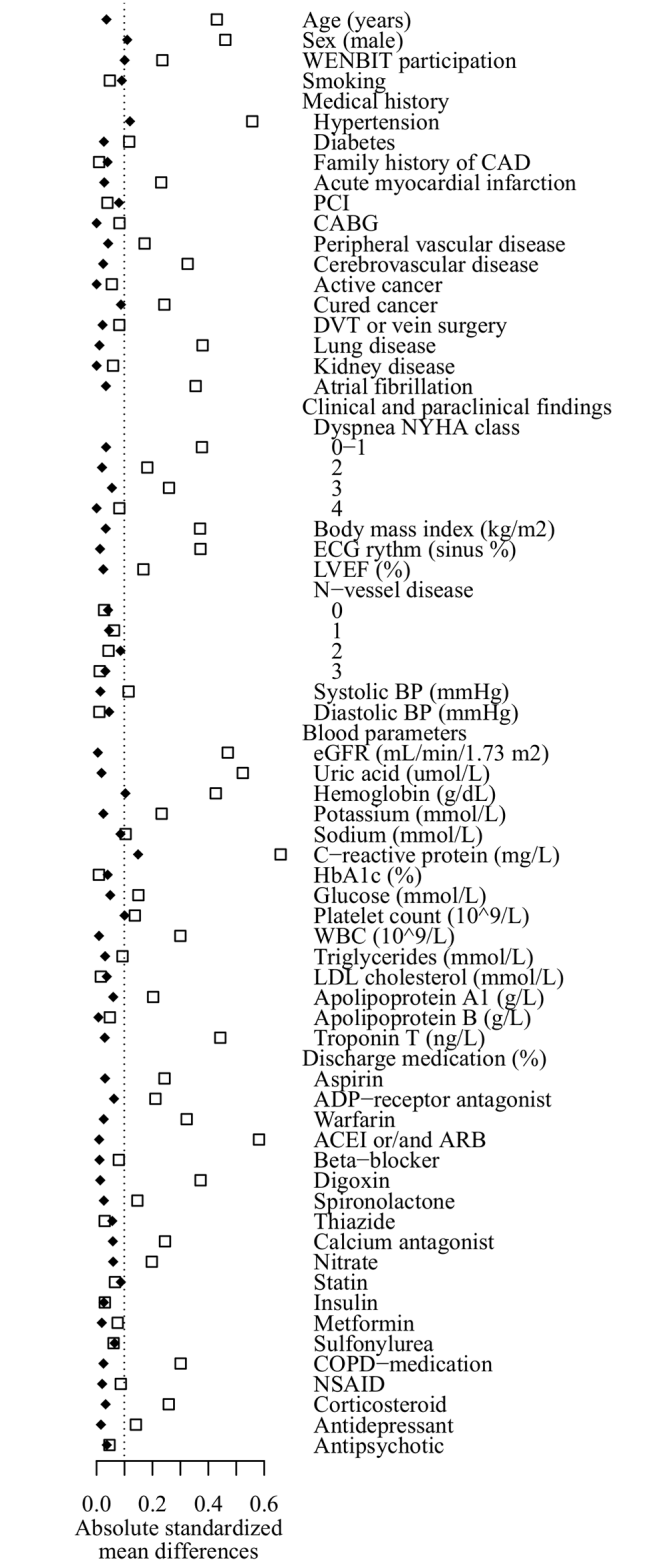
Absolute standardized mean differences for baseline characteristics before and after matching. Squares: Before matching. Diamonds: after matching.

Fig 1 displays the absolute standardized mean differences between all matching covariates before and after matching. Post matching, the differences for most covariates were ≤0.1, but somewhat higher for C-reactive protein (CRP) levels with a differences of 0.149. The propensity scores were evenly distributed in both groups (S1 and S2 Figs), and the ratios of variances of treated vs. controls were within the expected 95% CIs for equality for all continuous covariates, except for age with a ratio of 1.40 (S2 Table).

### Loop diuretics and all-cause mortality

**Fig 2:**
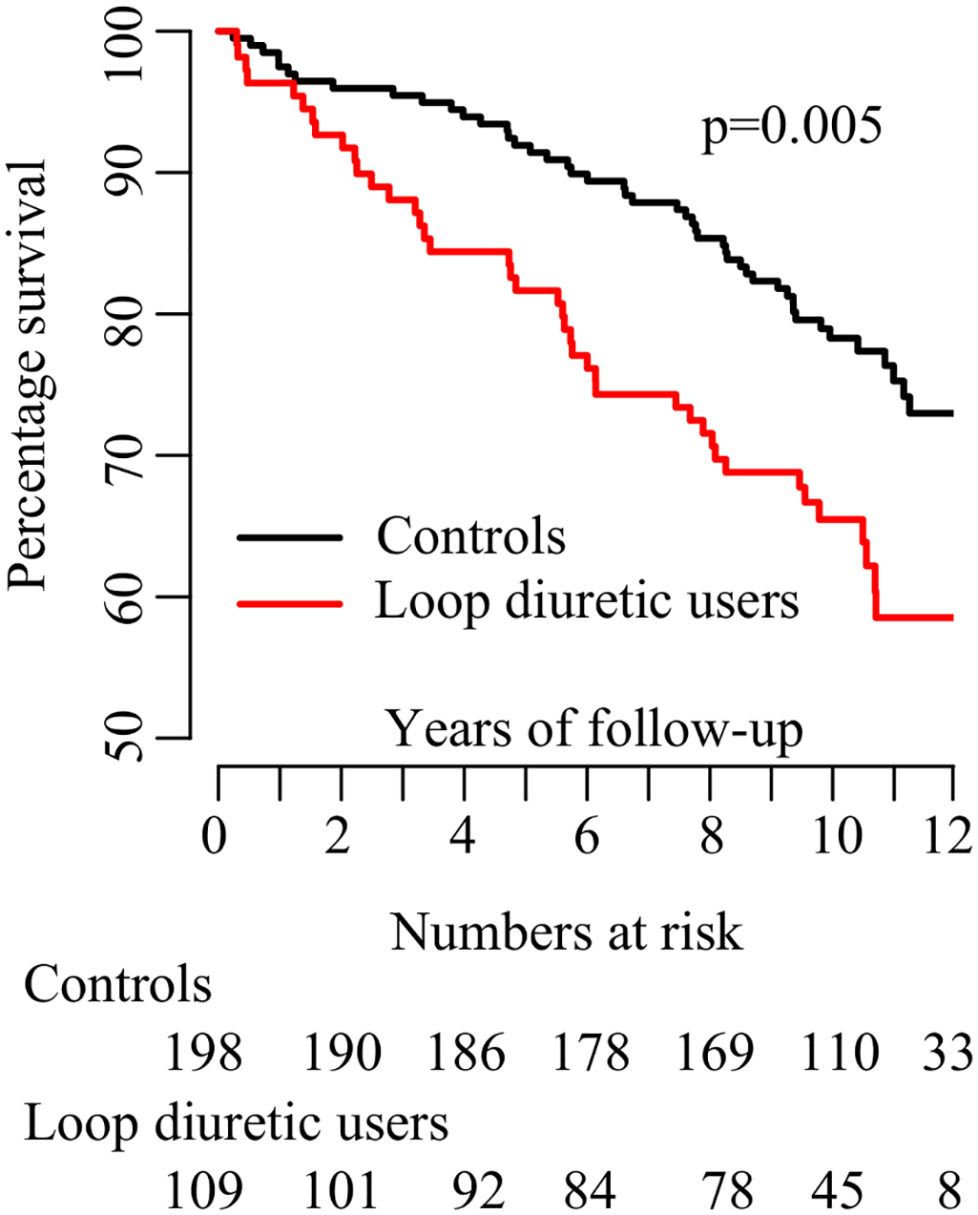
Kaplan-Meier plot showing survival curves for patients using loop diuretics and matched controls. The p-value for difference was calculated using log-rank test.

During a follow-up time of median (IQR) 10.1 (2.2) years, 41 (37.6%) of the treated patients died, as compared to 47 (23.7%) in the control group (p for log-rank test 0.005). Fig 2 shows a Kaplan Meier survival plot comparing the two groups. The unadjusted hazard ratio (HR) (95% CI) was 1.82 (1.20, 2.76). The number needed to harm was 7.2 (4.1, 30.3). Further adjusting for baseline covariates did not substantially attenuate the results (Table 2). Inclusion of all 3101 patients using propensity score weighting yielded similar estimates.

**Table 2:** Cox regression survival models.

				Unadjusted model		Adjusted model*	
Type of mortality	Years of follow up	N at risk	N events	Hazard ratio (95% CI)	P-value	Hazard ratio (95% CI)	P-value
**Propensity matched cohort**							
All-cause	10.1	307	88	1.82 (1.20, 2.76)	0.005	1.87 (1.15, 3.05)	0.011
CVD	10.1	307	40	1.55 (0.83, 2.90)	0.173	1.29 (0.59, 2.83)	0.52
Non-CVD	10.1	307	48	2.07 (1.18, 3.65)	0.012	2.01 (1.09, 3.71)	0.025
**Propensity weighted cohort**							
All-cause	10.4	3101	517	1.86 (1.58, 2.20)	<0.001	1.59 (1.32, 1.93)	<0.001
CVD	10.4	3101	207	2.12 (1.65, 2.71)	<0.001	1.69 (1.25, 2.29)	<0.001
Non-CVD	10.4	3101	310	1.69 (1.36, 2.11)	<0.001	1.47 (1.13, 1.89)	0.003

Abbreviations: CVD cardiovascular disease.

*Covariates: age, sex, study site, participation in the WENBIT study, smoking, family history of coronary heart disease, medical history (hypertension, diabetes, acute myocardial infarction, percutaneous intervention, coronary artery bypass surgery, peripheral vascular disease, cerebrovascular disease, cured cancer, active cancer (not in the cardiovascular mortality analyses due to failure of the model to converge), deep vein thrombosis or vein surgery, pulmonary disease, atrial fibrillation, dyspnea grade 0–4), measured parameters at baseline (body mass index, ECG rhythm, left ventricular ejection fraction, number of coronary vessels with >50% stenosis), laboratory values (estimated glomerular filtration rate, uric acid, hemoglobin, potassium, sodium, C-reactive protein, glycated hemoglobin, low density lipoprotein, troponin T), medication (aspirin, adenosine diphosphate receptor inhibitor, warfarin, angiotensin converting enzyme inhibitor and/or angiotensin receptor blocker, beta-blocker, digoxin, spironolactone, thiazide, calcium antagonist, nitrate, statin, insulin, metformin, sulfonylurea, treatment for chronic obstructive pulmonary disease, non-steroid anti-inflammatory drug, corticosteroid, antidepressant, antipsychotic), and baseline revascularization.

### Loop diuretics and cardiovascular and non-cardiovascular mortality

Cardiovascular death occurred in 17 (15.6%) patients using loop diuretics and in 23 (11.6%) of the controls (HR 1.55 (0.83, 2.90), p = 0.17). Correspondingly, non-cardiovascular deaths occurred in 24 (22.0%) and 24 (12.1%) patients (HR 2.07 (1.17, 3.65), p = 0.003). Adjustment for confounders resulted in similar point estimates.

### Sensitivity analysis

**Fig 3:**
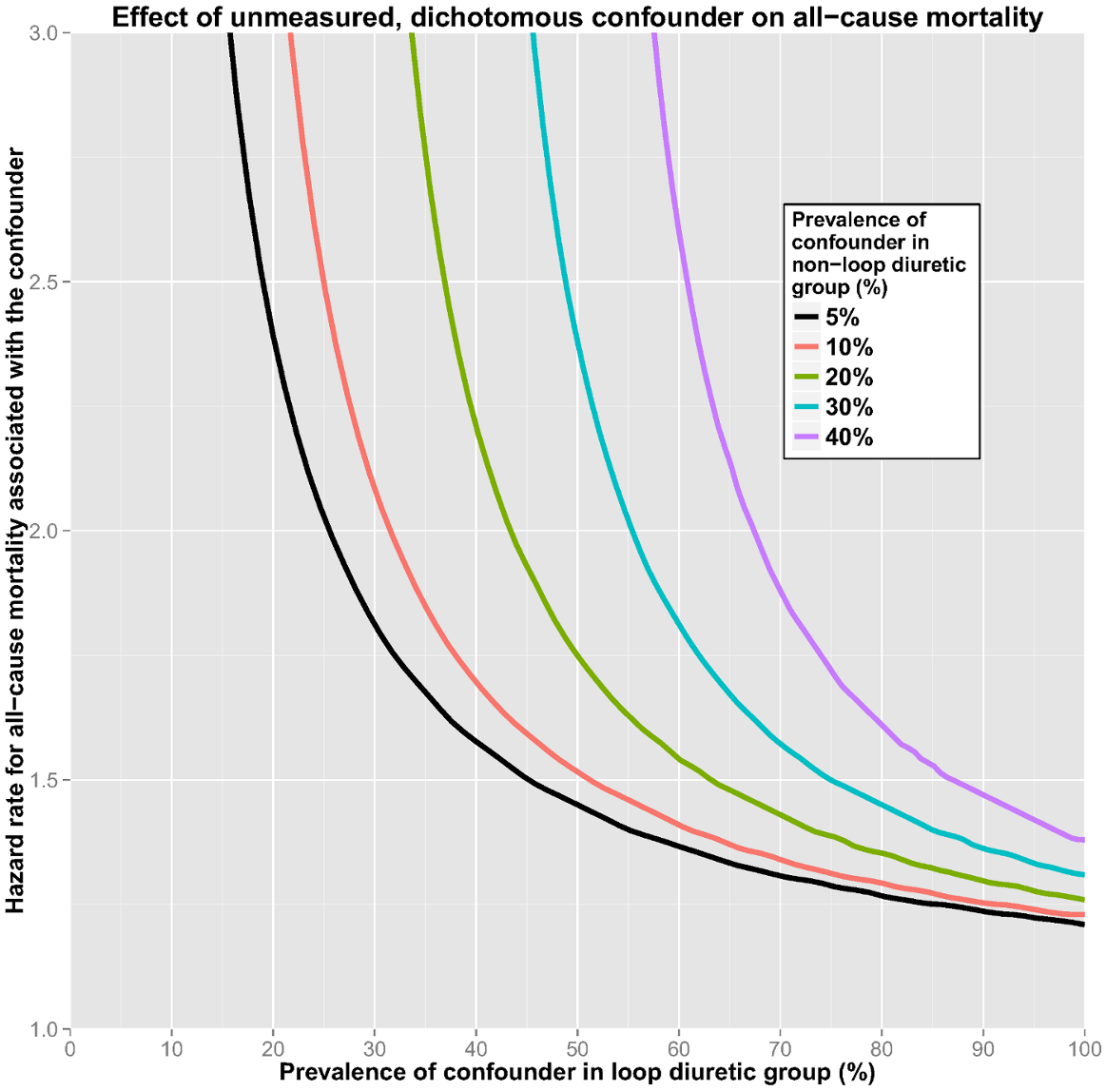
Sensitivity plot showing how high the hazard ratio of a single, unknown, dichotomous confounder would have to be, at different levels of distribution among patients using loop diuretics and controls, to fully explain the observed risk of death associated with loop diuretics.

Fig 3 shows how an unmeasured, dichotomous confounder could possibly explain the elevated risk associated with using loop diuretics. A single unmeasured confounder (e.g. frailty or diastolic heart failure), if present in 100% of the patients receiving the drug and merely 5% of the controls, could account for the observed risk if it was associated with a HR for all-cause mortality of about 1.2. However, a more realistic distribution of an unknown confounder would for example be 20% in the control group and 40% in the loop diuretic group, a situation in which the unknown confounder must confer a HR of 2.2.

## Discussion

In this observational, long term cohort of patients with suspected stable CAD, but without systolic heart failure or renal failure, subjects treated with loop diuretics had an almost twofold higher risk of all-cause death when compared to propensity matched controls. The number needed to harm was 7.2. Extensive adjustment did not substantially alter the results, and the findings were consistent also in the overall cohort using propensity score weighting.

### Existing evidence

Loop diuretics form the cornerstone in the treatment of systolic heart failure and hold strong recommendations in current guidelines [1, 2]. Yet, only a few, small RCTs investigating the impact of loop diuretics on survival in these patients have been conducted, the most recent almost 30 years ago [23–25]. The results were pooled in a meta-analysis with altogether 221 patients, including totally 15 fatalities during follow-up times ranging from a few weeks to one year, and showed significant improvement in survival [26]. In contrast, several large, more recent, observational studies have found increased mortality associated with the use of loop diuretics among patients with chronic systolic heart failure [6–9], also supported by a potential dose-response relationship [10–12]. In diastolic heart failure, a randomized pilot investigation in elderly patients found improvements in hemodynamic parameters three months after withdrawal of furosemide [27]. In acute heart failure the results from observational data are more ambiguous [28–30]. An RCT found no difference between a low-dose vs. high-dose strategy after 60 days [31], while a small RCT showed increased risk of adverse events in patients treated with continuous vs. bolus furosemide [32]. As for treatment of hypertension, a Cochrane review did not find sufficient evidence to recommend the use of loop diuretics, and noted the paucity of data available [4], whereas a recent observational study showed increased mortality in hypertensive patients with atrial fibrillation treated with loop diuretics [33]. In renal failure patients loop diuretics are frequently prescribed, but only low quality RCTs have been conducted and the effects are questionable [34]. Further, the drugs are also used to treat peripheral edema of various etiologies. A Dutch community based study reported high prevalence of “off label” use amongst elderly (16% for hypertension, 8% for peripheral edema and 8% for unknown reasons) [14].

Our results are coherent with earlier reports of increased mortality in chronic systolic heart failure patients treated with loop diuretics. The increased risk of non-cardiovascular mortality has, to our knowledge, not previously been described. Only one earlier study reported non-cardiovascular mortality and found no association with loop diuretics.[8]

### Possible pathomechanisms

Long-term use of loop diuretics may lead to a plethora of possible adverse effects. Importantly, furosemide, the most commonly used substance, has a very erratic absorption [35]. The risk of hypokalemia, associated with potentially lethal arrhythmias, is well recognized [36], and potassium supplementation to loop diuretics is associated with improved survival [37]. In the elderly, hyponatremia and hypovolemia are common side effects, being related to several outcomes which again are associated with poor survival, such as osteoporosis, hypotension, confusion and brain damage [13]. Polypharmacy is becoming increasingly prevalent, and loop diuretics carry a significant risk of drug-drug interactions [38]. They are sometimes combined with a thiazide to treat diuretic resistance, albeit with the risk of severe side effects [39]. With the emergence of combination pills to treat hypertension, this might also happen unintentionally [13]. It is interesting to note the potential paradoxical effect of loop diuretics in systolic heart failure; increased diuresis leads to hypovolemia which induces renin-angiotensin system activation and sympathetic stimulation [40]—pathways which when blocked are the only proven way to reduce systolic heart failure mortality. Accordingly, an increased risk of mortality was found in rats randomized to furosemide, compared to placebo or the combination of furosemide and ramipril [41].

Higher risk of renal cell carcinoma is described in patients treated with loop and thiazide diuretics [42], and furosemide-induced DNA damage has been observed in mice [43]. However, to our knowledge, no clear association between loop diuretics and carcinogenesis has been established. Given the increased risk of both cardiovascular and non-cardiovascular mortality, one can speculate that the sum of circulatory and metabolic changes caused by loop diuretics might aggravate the course of other diseases, irrespective of etiology. However, such adverse effects likely must have developed during follow-up, given the extensive baseline matching.

### Strengths and limitations

When analysing a single baseline variable, propensity score matching is one of the most robust ways of approaching observational data in order to reduce confounding and assess possible causality [44]. In this study, acceptable balance between treated and controls was achieved, according to several tests [19]. Only CRP was slightly higher in treated patients. Consistency across both different propensity score methods and model selections support the potential of a true causal relationship. Regardless of rigorous statistical efforts, residual confounding almost certainly exists. However, sensitivity analyses show that such a confounder (or group of confounders) must either be almost perfectly asymmetrically distributed between the groups or confer a very high HR for mortality if it were to explain the findings by confounding alone.

An important limitation of the current work is the lack of information about left ventricular diastolic function and clinical signs of fluid overload. We did, however, take into account conditions strongly associated with diastolic heart disease, such as obesity, diabetes, hypertension, age and atrial fibrillation. Other limitations include the lack of data on the duration of treatment prior to study inclusion, the type and dose of loop diuretic, and drug compliance during follow-up.

### Clinical implications

No long-term RCTs of loop diuretics have been conducted. Hence, this and other recent observational studies provide the best current evidence base and suggest potential harm by such treatment [6–12]. Importantly, due to the lack of RCTs, the evidence needed to reject the hypothesis of clinical benefit is less strict than would otherwise be the case. According to the maxim ‘primum non nocere’ our study argues against the use of loop diuretics in patients without a strong indication. Indeed, the recently coined term ‘Morbus Diureticus’ [13], used to describe the perils of epidemic overuse of diuretics in the elderly, may be eerily accurate.

### Conclusions

In patients with suspected coronary artery disease but without systolic heart failure or renal failure, use of loop diuretics is associated with all-cause mortality. In such patients, without clinical signs of fluid overload, discontinuation of loop diuretics should be considered.

## Supporting Information

S1 Fig Jitter plots of the distributions of propensity scores before and after matching among patients treated with loop diuretics (treatment units) and controls, illustrating which patients were excluded from the final analyses due to inadequate matching. (TIF)

S2 Fig Histograms displaying the distribution of propensity scores before (raw) and after matching among patients treated with loop diuretics (treated) and controls. (TIF)

S1 Table Baseline characteristics before matching. (DOCX)

S2 Table Variance ratios of continuous variables between patients receiving loop diuretics and controls. (DOCX)
