# Supplementary figures and images for: Use of Loop Diuretics is Associated with Increased Mortality in Patients with Suspected Coronary Artery Disease, but without Systolic Heart Failure or Renal Impairment: An Observational Study Using Propensity Score Matching
Source: PLoS One. 2015 Jun 1;10(6):e0124611. doi: 10.1371/journal.pone.0124611 (PMC4452510; doi:10.1371/journal.pone.0124611)

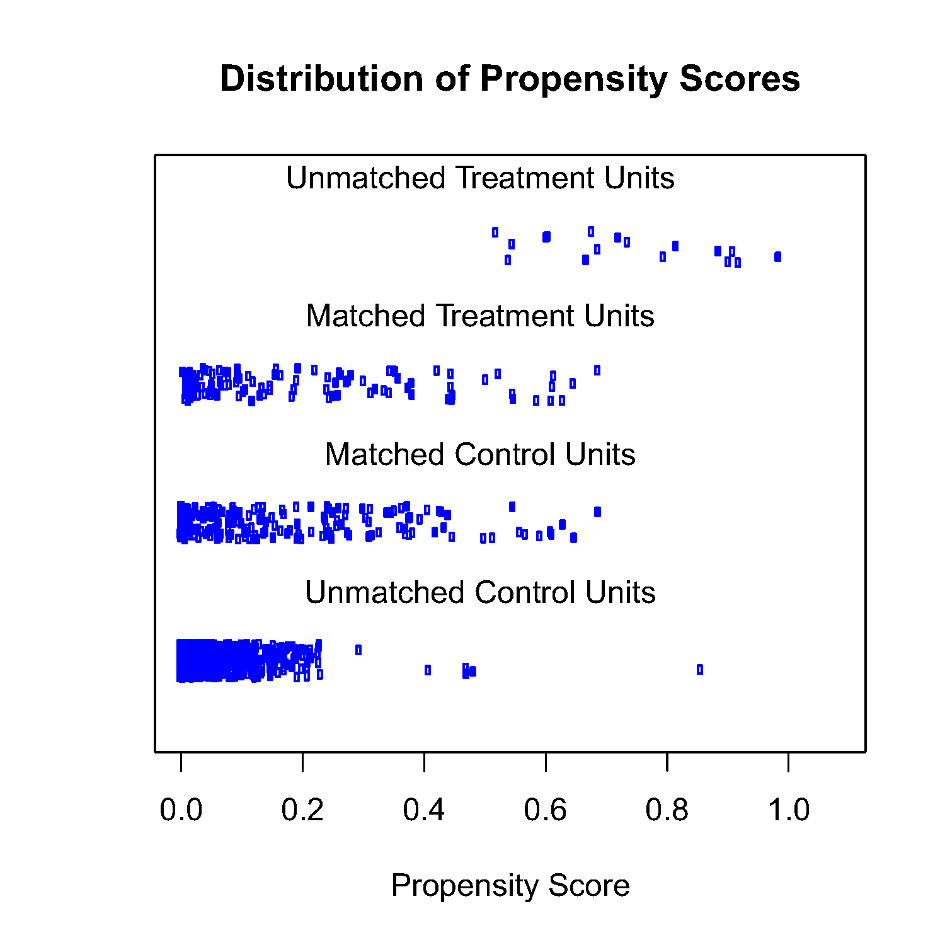

Supplement: S1 Fig — (TIF) [file pone.0124611.s001.tif]

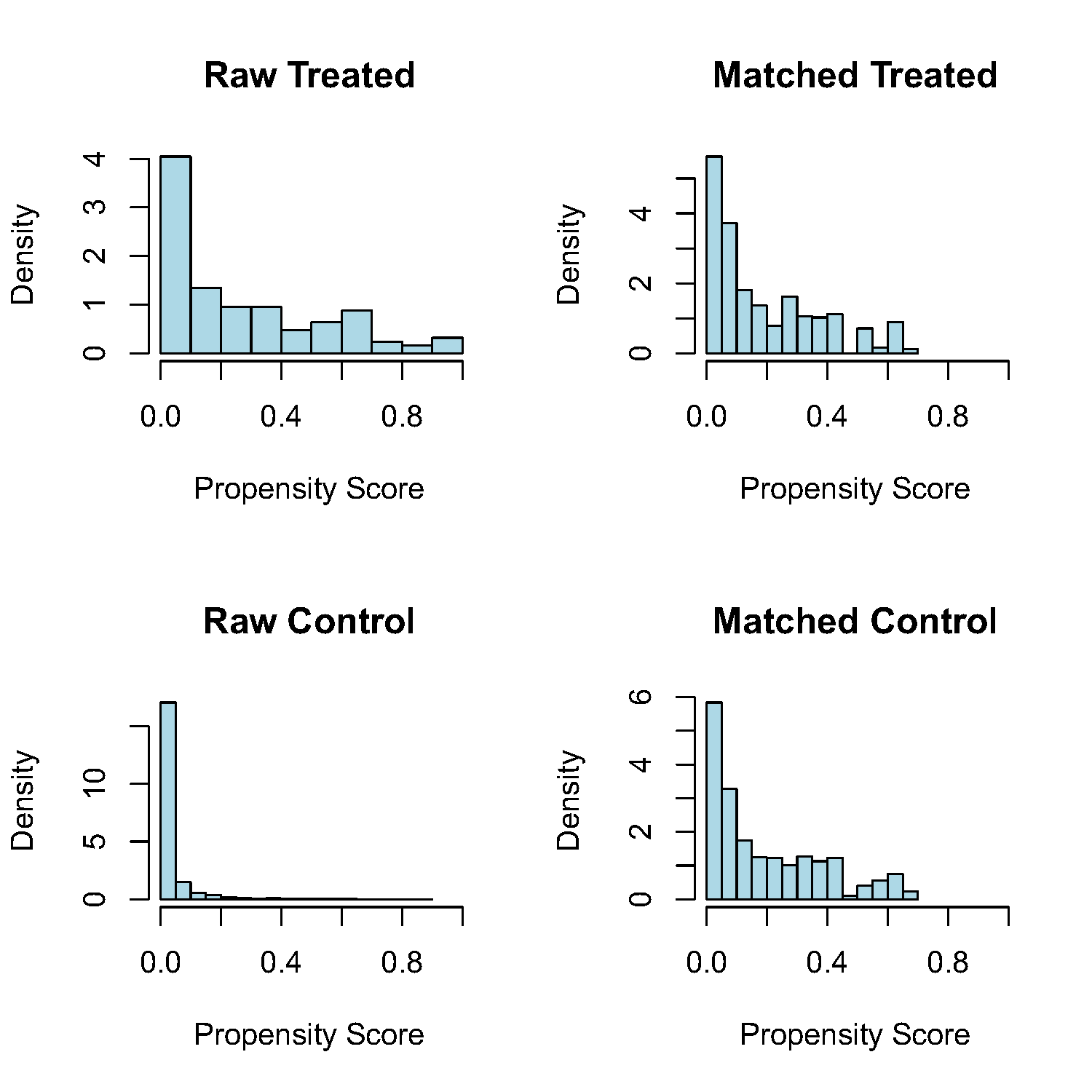

Supplement: S2 Fig — (TIF) [file pone.0124611.s002.tif]
