# Supplementary material for: Use of Loop Diuretics is Associated with Increased Mortality in Patients with Suspected Coronary Artery Disease, but without Systolic Heart Failure or Renal Impairment: An Observational Study Using Propensity Score Matching
Source: PLoS One. 2015 Jun 1;10(6):e0124611. doi: 10.1371/journal.pone.0124611 (PMC4452510; doi:10.1371/journal.pone.0124611)
Supplement: S1 Table — (DOCX) [file pone.0124611.s003.docx]

| **S1 Table. Baseline characteristics before matching** | | | | | | |
| --- | --- | --- | --- | --- | --- | --- |
|  | | Controls |  | Loop diuretics |  | P-value |
| N | | 2975 |  | 126 |  |  |
| Age (years) | | 60.5 (10) |  | 65.1 (11) |  | <0.001 |
| Sex (male %) | | 2152 (72.3) |  | 62 (49.2) |  | <0.001 |
| WENBIT participation (%) | | 1839 (61.8) |  | 63 (50) |  | 0.01 |
| Smoking (%) | | 938 (31.5) |  | 37 (29.4) |  | 0.68 |
| *Medical history (%)* | |  |  |  |  |  |
| Hypertension | | 1314 (44.2) |  | 88 (69.8) |  | <0.001 |
| Diabetes | | 303 (10.2) |  | 18 (14.3) |  | 0.18 |
| Family history of CAD | | 957 (32.2) |  | 40 (31.7) |  | 1.00 |
| Acute myocardial infarction | | 1002 (33.7) |  | 57 (45.2) |  | 0.01 |
| PCI | | 544 (18.3) |  | 25 (19.8) |  | 0.75 |
| CABG | | 275 (9.2) |  | 15 (11.9) |  | 0.40 |
| Peripheral vascular disease | | 207 (7) |  | 16 (12.7) |  | 0.02 |
| Cerebrovascular disease | | 167 (5.6) |  | 23 (18.3) |  | <0.001 |
| Active cancer | | 46 (1.5) |  | 3 (2.4) |  | 0.71 |
| Cured cancer | | 103 (3.5) |  | 14 (11.1) |  | <0.001 |
| DVT or vein surgery | | 90 (3) |  | 6 (4.8) |  | 0.40 |
| Pulmonary disease | | 319 (10.7) |  | 35 (27.8) |  | <0.001 |
| Kidney disease | | 8 (0.3) |  | 1 (0.8) |  | 0.82 |
| Atrial fibrillation | | 203 (6.8) |  | 27 (21.4) |  | <0.001 |
| *Clinical and paraclinical findings* | |  |  |  |  |  |
| Dyspnea (NYHA class) (%) | |  |  |  |  |  |
|  | 0-1 | 2117 (71.2) |  | 67 (53.2) |  | <0.001 |
|  | 2 | 736 (24.7) |  | 42 (33.3) |  | 0.04 |
|  | 3 | 120 (4) |  | 16 (12.7) |  | <0.001 |
|  | 4 | 2 (0.1) |  | 1 (0.8) |  | 0.27 |
| Body mass index (kg/m2) | | 26.7 (3.8) |  | 28.8 (5.6) |  | <0.001 |
| ECG rythm (sinus%) | | 2893 (97.2) |  | 105 (83.3) |  | <0.001 |
| LVEF (%) | | 67 (7.7) |  | 65.5 (8.8) |  | 0.07 |
| Angiographic extent of CAD (%) | |  |  |  |  |  |
|  | 0-vessel disease | 837 (28.1) |  | 37 (29.4) |  | 0.84 |
|  | 1-vessel disease | 714 (24) |  | 27 (21.4) |  | 0.58 |
|  | 2-vessel disease | 654 (22) |  | 30 (23.8) |  | 0.71 |
|  | 3-vessel disease | 770 (25.9) |  | 32 (25.4) |  | 0.99 |
| Systolic BP (mmHg) | | 142 (20) |  | 144 (21) |  | 0.21 |
| Diastolic BP (mmHg) | | 81.6 (10) |  | 81.7 (11) |  | 0.91 |
| *Blood parameters* | |  |  |  |  |  |
| eGFR (mL/min/1.73 m2) | | 91.3 (13) |  | 84.5 (14) |  | <0.001 |
| Uric acid (umol/L) | | 346 (78) |  | 396 (95) |  | <0.001 |
| Hemoglobin (g/dL) | | 14.4 (1.2) |  | 13.8 (1.3) |  | <0.001 |
| Potassium (mmol/L) | | 4.31 (0.31) |  | 4.23 (0.32) |  | 0.01 |
| Sodium (mmol/L) | | 142 (2.3) |  | 142 (2.3) |  | 0.26 |
| C-reactive protein (mg/L) | | 1.64 (2.5) |  | 3.32 (4.4) |  | <0.001 |
| HbA1c (%) | | 6.19 (1.4) |  | 6.18 (1.2) |  | 0.93 |
| Glucose (mmol/L) | | 248 (69) |  | 258 (74) |  | 0.13 |
| Platelet count (10^9/L) | | 6.26 (2.3) |  | 6.56 (2) |  | 0.10 |
| WBC (10^9/L) | | 7.06 (2) |  | 7.65 (2) |  | 0.001 |
| Triglycerides (mmol/L) | | 1.5 (1.1) |  | 1.44 (1) |  | 0.52 |
| Low density lipoprotein (mmol/L) | | 3.09 (0.98) |  | 3.1 (1.1) |  | 0.87 |
| Apolipoprotein A1 (g/L) | | 1.33 (0.27) |  | 1.39 (0.3) |  | 0.03 |
| Apolipoprotein B (g/L) | | 0.898 (0.24) |  | 0.909 (0.23) |  | 0.60 |
| Troponin T (ng/L) | | 4 (5) |  | 7 (12) |  | <0.001 |
| *Discharge medication (%)* | |  |  |  |  |  |
| Aspirin | | 2453 (82.5) |  | 90 (71.4) |  | 0.002 |
| ADP-receptor inhibitor | | 468 (15.7) |  | 12 (9.5) |  | 0.08 |
| Warfarin | | 104 (3.5) |  | 19 (15.1) |  | <0.001 |
| ACEI or/and ARB | | 740 (24.9) |  | 68 (54) |  | <0.001 |
| Beta-blocker | | 2116 (71.1) |  | 94 (74.6) |  | 0.46 |
| Digoxin | | 36 (1.2) |  | 18 (14.3) |  | <0.001 |
| Spironolactone | | 18 (0.6) |  | 4 (3.2) |  | 0.005 |
| Thiazide | | 210 (7.1) |  | 8 (6.3) |  | 0.90 |
| Calcium antagonist | | 647 (21.7) |  | 42 (33.3) |  | 0.003 |
| Nitrate | | 648 (21.8) |  | 39 (31) |  | 0.02 |
| Statin | | 2373 (79.8) |  | 97 (77) |  | 0.52 |
| Insulin | | 79 (2.7) |  | 4 (3.2) |  | 0.94 |
| Metformin | | 134 (4.5) |  | 8 (6.3) |  | 0.45 |
| Sulfonylurea | | 103 (3.5) |  | 6 (4.8) |  | 0.60 |
| COPD-medication | | 161 (5.4) |  | 21 (16.7) |  | <0.001 |
| NSAID | | 106 (3.6) |  | 7 (5.6) |  | 0.35 |
| Corticosteroid | | 42 (1.4) |  | 11 (8.7) |  | <0.001 |
| Antidepressant | | 160 (5.4) |  | 12 (9.5) |  | 0.07 |
| Antipsychotic | | 30 (1) |  | 2 (1.6) |  | 0.86 |
| Continuous variables are shown as means (standard deviation) and medians (interquartile range) and categorical variables as numbers (percentage).  Abbreviations: WENBIT=WEstern Norway B-vitamin Trial; CAD=coronary artery disease; PCI=percutaneous coronary intervention; CABG=coronary artery bypass graft; PVD=peripheral vascular disease; DVT=deep venous thrombosis; NYHA=New York Heart Association; BP=blood pressure; eGFR=estimated glomerular filtration rate; HbA1c=glycated hemoglobin; WBC=white blood cell count; ACEI=angiotensin converting enzyme inhibitor; ARB=angiotensin receptor blocker; COPD=chronic obstructive pulmonary disease; NSAID=non-steroid anti-inflammatory drug | | | | | | |
