# Supplementary material for: Use of Loop Diuretics is Associated with Increased Mortality in Patients with Suspected Coronary Artery Disease, but without Systolic Heart Failure or Renal Impairment: An Observational Study Using Propensity Score Matching
Source: PLoS One. 2015 Jun 1;10(6):e0124611. doi: 10.1371/journal.pone.0124611 (PMC4452510; doi:10.1371/journal.pone.0124611)
Supplement: S2 Table — (DOCX) [file pone.0124611.s004.docx]

| **S2 Table.** **Variance ratios of continuous variables between patients receiving loop diuretics and controls** | | | |
| --- | --- | --- | --- |
|  | Before matching |  | After matching |
| Age | 1.12 |  | 1.40 |
| Body mass index | 2.16 |  | 0.89 |
| Left ventricular ejection fraction | 1.29 |  | 1.18 |
| Systolic blood pressure | 1.10 |  | 1.15 |
| Diastolic blood pressure | 1.20 |  | 1.14 |
| Estimated glomerular filtration rate | 1.20 |  | 1.26 |
| Uric acid | 1.47 |  | 1.27 |
| Hemoglobin | 1.19 |  | 1.04 |
| Potassium | 1.06 |  | 0.99 |
| Sodium | 0.98 |  | 0.87 |
| C-reactive protein | 0.98 |  | 0.91 |
| Glycated hemoglobin | 0.77 |  | 0.90 |
| Glucose | 0.79 |  | 0.78 |
| Platelet count | 1.12 |  | 0.80 |
| White blood cell count | 0.95 |  | 1.21 |
| Triglycerides | 0.95 |  | 0.91 |
| Low density lipoprotein | 1.15 |  | 1.27 |
| Apolipoprotein A1 | 1.25 |  | 1.03 |
| Apolipoprotein B | 0.96 |  | 1.03 |
